# Does near-infrared (NIR) fluorescence angiography modify operative strategy during emergency procedures?

**DOI:** 10.1007/s00464-018-6226-9

**Published:** 2018-05-16

**Authors:** Emilie Liot, Michela Assalino, Nicolas Christian Buchs, Boris Schiltz, Jonathan Douissard, Philippe Morel, Frédéric Ris

**Affiliations:** 1 Unit of Visceral and Transplant Surgery, Department of Surgery, Geneva University Hospitals and Medical School, Geneva, Switzerland; 2 Department of Surgery, Service of Visceral Surgery, Geneva University Hospitals, Rue Gabrielle Perret-Gentil 4, 1211 Geneva, Switzerland

**Keywords:** Near-infrared (NIR) angiography, Emergency surgery, Modification of operative strategy, Organ viability, Assessment of intestinal perfusion, Limits of intestinal resection

## Abstract

**Introduction:**

Bowel viability can be difficult to evaluate during emergency surgery. Near-infrared (NIR) fluorescence angiography allows an intraoperative assessment of organ perfusion during elective surgery and might help to evaluate intestinal perfusion during emergency procedures. The aim of this study was to assess if NIR modified operative strategy during emergency surgery.

**Materials and methods:**

From July 2014 to December 2015, we prospectively evaluated all consecutive patients, who had NIR assessment during emergency surgery. Primary endpoint was the modification of operative strategy after the assessment with NIR. Secondary endpoints were general post-operative outcomes, including reoperation rate.

**Results:**

Fifty-six patients were included in the study. Mean age was 64 ± 17 years. An exploratory laparoscopy was performed in 39% (*n* = 22) and an open surgery in 61% of cases (*n* = 34). Conversion rate to open surgery was 41% (*n* = 9). 32 patients had a bowel resection. In 32% of the cases (*n* = 18), the result of the NIR test led to a modification of the operative strategy. Among them, 33% (*n* = 6) had a larger resection or a resection, which was initially not planned. The other 12 patients (67%) had finally no resection, which was initially thought to be performed. Importantly, none of those patients needed a reoperation for ischemia. Mean time for performing NIR test was 167 s (± 121). Overall reoperation rate was 16.1% (*n* = 9). Two patients had an anastomotic leak. Eight patients (14.3%) died within the first 30 post-operative days; however, none of them presented a bowel ischemia or an anastomotic leak.

**Conclusion:**

NIR is an easy and short procedure, which can be performed during emergency surgery to assess bowel perfusion. It may help the surgeon to preserve intestinal length or to define the exact limits of resection. Overall, we report a modification of operative strategy in up to one-third of evaluated patients.

**Electronic supplementary material:**

The online version of this article (10.1007/s00464-018-6226-9) contains supplementary material, which is available to authorized users.

During emergency abdominal procedures, one of the main goals is to have a simple, quick and reliable evaluation of the organs viability in order to decide whether a resection is necessary or not. On one hand, unnecessary resection should be avoided because it increases surgical time as well as post-operative morbidity. On the other hand, leaving a non-viable organ or performing an insufficient resection can lead to major complications such as organ necrosis, infections, anastomosis leakage, reoperations, or even death. On-call surgeons need to make a quick but often difficult decision, especially if they are in training.

An adequate blood supply is an essential condition to ensure organ viability. To assess the perfusion, several methods have been described. In 2011, Urbanavicius et al. [1] published a review of those techniques. Traditionally, surgeons based their decision on clinical assessment: organ color, presence of bowel peristalsis, evaluation of pulsation, and bleeding from the marginal arteries. In addition, intraoperative Doppler ultrasound of the vessels can be performed. However, clinical qualitative judgment remains subjective and a large number of factors may alter this perception. Hypotension, noradrenergic drugs or spasm could influence the detection of pulsation in viable organs. Furthermore, it has been proven that non-vascularized bowel continues to have peristaltic movements [2]. Many techniques are difficult to apply in a daily practice, while others are not accurate enough. In 2015, Ris et al. described some of the new techniques available to assess bowel perfusion in elective colorectal surgery. Among them, Near-infrared fluorescence (NIR) angiography, using indocyanine green (ICG), seemed to be one of the most promising tools [3].

This technology requires a special endoscope emitting a signal at a wavelength of 805 nm to visualize the blood flow in vessels and organs [4, 5]. NIR angiography can be performed both during laparoscopic and open surgery with the same material and repeated as often as necessary with a ICG limit of 2 mg/kg.

Actually, this technique offers much more applications to the general surgeon, including help during liver resection, ureteral localization [6], or abdominal wall reconstructive surgery as summarized by Gossedge et al. [7].

However, NIR fluorescence angiography during emergency abdominal procedures has been rarely used and poorly studied. The studies were often done on animal models [8, 9]. Some rare series described its relevance to determinate the viability of bowel and to identify the limits of bad perfused segments in patients with ischemic disease [10], but included only a small number of cases [6, 11–13].

The aim of this study is to assess if NIR angiography can be used to modify the operative strategy during emergency procedures.

## Materials and methods

From July 2014 to December 2015, we performed a retrospective study of prospective collected data, including all adult patients who had a NIR evaluation during an abdominal emergency surgery at our institution. The study complies with local ethical standards. All participating subjects provided oral informed consent for the use of their data for research. Since this is a retrospective evaluation of data, which were collected during emergency procedures, it is exempt from ethics committee. Patients who underwent emergency surgery without NIR angiography were excluded from the study.

Primary endpoint was the impact of NIR, on planned operative strategy during emergency surgery.

Secondary endpoints included the indications for the use of NIR test during emergencies as well as post-operative outcomes. Complications were graded according to the Clavien–Dindo classification [14].

### Surgical procedure

The choice of the surgical approach was at the discretion of the on-call surgeon. At his/her request, a standard dose of 7.5 mg of ICG (3 ml of 2.5 mg/ml aqueous solution) was administrated by a venous injection followed by a flush of 10 ml of 0.9% saline solution. A pinpoint endoscopic fluorescence imaging system was used (Novadaq, Vancouver, Canada) for fluorescence imaging.

Time from the injection to the neo-infrared signal onset, total time of the NIR test (measured from request of ICG injection to decision making) as well as clinical parameters (blood pressure, heart frequency, oxygen saturation, and amine support) were prospectively recorded.

The surgeon was free to choose indications and timing for the NIR angiography. If deemed necessary, more than one angiography was realized during the same surgical procedure.

A modification of operative strategy was defined as any deviation from the initial strategy, according to the result of NIR test.

All intraoperative data were recorded on a standardized data collection set. Perioperative characteristics and outcomes were recorded and analyzed.

## Results

A total of 56 consecutive patients were included in the present series.

### Patients characteristics

Twenty-seven males (48.2%) and twenty-nine females (51.8%) were included, with a mean age of 64 years (± 17 years). Median Body Mass Index was 25.8 kg/m^2^ (± 6.5 kg/m^2^). Almost half of the patients (46.2%) had an American Society of Anesthesiologists (ASA) score upper or equal to 3.

Patients characteristics are summarized in Table 1.

**Table 1:** Patients characteristics

Number of patients	56
Male	27 (48.2%)
Female	29 (51.8%)
Mean age at operation time (years) ± SD	4.3 ± 17
ASA score	
I	1 (1.8%)
II	28 (50%)
III	14 (25%)
IV	12 (21.4%)
V	1 (1.8%)
Mean BMI (Kg/m^2^) ± SD	25.8 ± 6.5
Other risk factors	
Cardiovascular history	17 (30.4%)
Smoking	14 (25%)
Diabetes	4 (7.1%)
Inflammatory bowel disease	3 (5.4%)

### Operation characteristics

Surgical indications are summarized in Table 2. The most frequent pathology was intestinal obstruction (adhesions, tumor, hernia). Of note, seven patients had a bowel ischemia.

**Table 2:** Perioperative data

Indication for surgery	
Occlusive tumor	12 (21.5%)
Perforation	4 (7.1%)
Adhesions or internal hernia	18 (32.1%)
Mesenteric ischemia	7 (12.5%)
Groin hernia	5 (8.9%)
Diverticulitis	2 (3.6%)
Others^a^	8 (14.3%)
Type of surgical approach	
Laparoscopy	22 (39.2%)
Laparotomy	34 (60.8%)
Conversion rate	9 (41%)
Type of surgery	
Exploration and adhesiolysis	24 (42.9%)
Small bowel resection	7 (12.5%)
Colon resection	27 (48.2%)
Ileostomy	7 (12.5%)
Colostomy	8 (14.3%)
Anastomosis	20 (35.7%)
Other procedures^b^	15 (37.5%)
Type of disease	
Cancer	15 (26.8%)
Benign disease	41 (73.2%)

^a^Mesenteric hematoma, appendicitis, inflammatory pseudotumor, segmental hepatic necrosis, symptomatic colon cancer, coeliac trunk dissection, mesenteric abscess

^b^Hernia repair, hepatectomy, splenectomy, gynecological resection, negative pressure wound therapy

Laparoscopic approach was performed in 39% (*n* = 22) with a conversion rate of 41% (*n* = 9). Conversion was necessary in eight cases for bowel resection and in one case for hernia reduction. No conversion was required to realize the NIR test.

Open surgery was performed in 61% (*n* = 34) of the cases.

Bowel resection was performed in 32 patients (57.1%). Most of them had a colonic resection (*n* = 25 vs. *n* = 5 for small bowel resection) and two patients had a synchronous colonic and small bowel resection. Among those patients, twenty (62.5%) patients had a direct anastomosis and 12 (32.5%) had a stoma (ileostomy or colostomy). One hepatectomy, one splenectomy for ischemia, and three gynecological resections for oncological reasons were necessary as well.

Pathology reports showed an intra-abdominal neoplasia in 26.8% of all cases (*n* = 15).

Surgical procedures were performed by 17 different on-call surgeons with various levels of surgical experience: half of them (*n* = 28) were done by junior surgeons under supervision.

In 76.8% of cases (*n* = 43) NIR angiography was used once, and in 23.2% of cases (*n* = 13) NIR was used twice.

Mean time of the overall NIR test was 167 s (± 121 s) with a mean time to the onset of the signal of 30 s (± 19 s).

### Modification of operative strategy

In 32% (*n* = 18), the NIR angiography led to a modification of the surgical decision. Among them, 67% (*n* = 12) had a more conservative strategy (Images 1 and 2 in Supplementary material 1 and 2). Indeed, no resection was finally performed, while initially thought. In addition, 33% of the patients with a modification in operative strategy (*n* = 6) required a larger resection or a resection which was not initially planned.

There was no change of attitude after NIR test in 68% (*n* = 38).

**Fig. 1:**
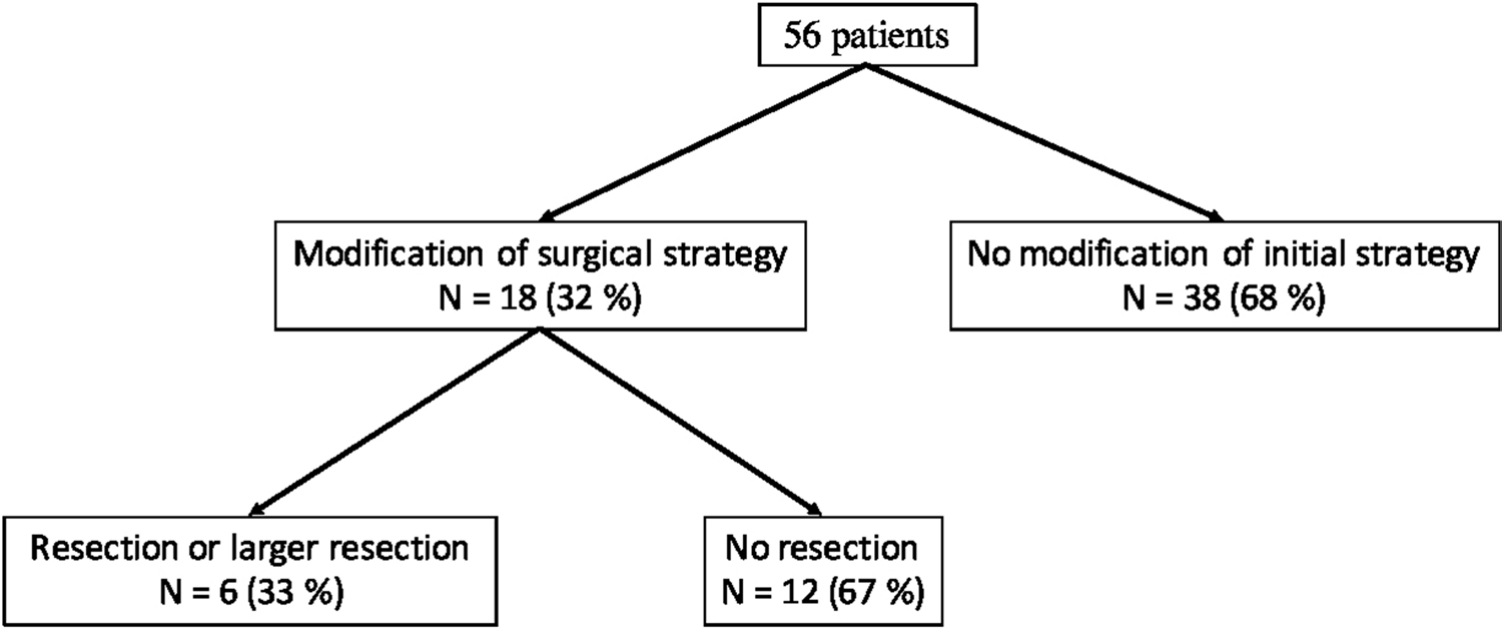
Study flowchart

The study flowchart is described in Fig. 1.

### Indications for NIR test

Most of the decision to use the near infrared was to assess organ viability (50%, *n* = 28). In those situations, evaluation almost exclusively focused on the bowel tract: 55% for small bowel, 39% for the colon, and 2% for stomach. In only 4% of cases, NIR angiography was performed to assess the perfusion of the spleen or of the liver.

Among the 32 patients who had a bowel resection, NIR angiography was performed at various times of the surgical procedures: before section of the bowel to control the vascularization of the pre-selected area (5%, *n* = 3) (Image 3 in Supplementary material 3), after section in order to control stump perfusion (34%, n = 19) and/or to check the quality of the anastomosis perfusion (18%, *n* = 10). Of note, only 50% of the anastomosis were assessed.

### Reoperation rate

Overall reoperation rate within 30 days was 16.1% (*n* = 9). Four patients with negative pressure wound therapy had a planned second look and dressing change without resection. Three patients with significant deterioration in the general condition required a reoperation: one needed a splenectomy, one an adhesiolysis, and the last one developed an abdominal compartment syndrome after right hemicolectomy for ischemia without anastomosis. No additional resection was necessary.

Finally, 2 out of the 20 anastomoses (10%) had an anastomotic leakage and needed a reoperation. One leak occurred 11 days after a subtotal colectomy with ileo-sigmoid anastomosis for obstructive metastatic cancer of the splenic flexure (Adenocarcinoma pT4a pN2a M1 R0). The second anastomotic dehiscence occurred on day 10 after a colorectal anastomosis for obstructive sigmoid cancer with peritoneal carcinomatosis (Adenocarcinoma pT4a N2b M1b R2) in a patient with severe malnutrition.

In both situations, NIR perfusion of organs was good and the test did not change the management of those patients. In the first case, the test was performed after bowel division, before anastomosis; and in the second case, angiography was done twice during the initial surgery: before large bowel resection and after anastomosis.

### Mortality rate

30-day mortality rate was 14.3% (*n* = 8). All these patients had an ASA score of 4 at the time of surgery. Six patients died of multiorgan failure (four patients during the first 48 h, one patient at day 19, and another at day 22), one patient died of cardiogenic shock and one cirrhotic patient died of gastrointestinal perforation at day 26 probably independent of the initial vascularization assessment. The patient complained indeed about acute abdominal pain after ascites puncture and a CT scanner confirmed the presence of a new pneumoperitoneum. A reoperation was not performed because of the bad general condition of the patient.

Interestingly, the polytrauma patient with ASA 5 score at time of surgery had a suspicion of gastric and spleen necrosis after coeliac trunk dissection. NIR angiography confirmed the spleen necrosis and the indication for splenectomy. However, stomach perfusion was good and the organ could be spared. After two months at Intensive Care Unit, the patient was transferred to another center.

No death was due to new bowel ischemia or anastomotic leakage.

## Discussion

In our study, we evaluated the utility of NIR angiography during unselected emergency abdominal surgery involving bowel ischemia, aiming to determine if the use of this technology alter the surgery and could help decision making during emergency procedures.

Results of the NIR test led to a change in attitude in 32% of the cases. Interestingly, in two-third of these situations, operative strategy was changed for a less aggressive approach, while in the other third group, we had to perform a larger resection than initially planned. None of those patients had a reoperation for ischemia. Therefore, the use of NIR allowed to spare tissues and organs, and avoid complications due to insufficient resections. This confirms our feeling that the technology is a promising tool to assess quickly and simply bowel perfusion.

Furthermore, in this study, reoperation and 30-day mortality rates of respectively 16.1% and 14.3% are comparable to the literature when NIR angiography was not performed. Recently, Nielsen et al. reported a reoperation rate between 17 and 20% and a mortality rate of 8.3–22.4% in a large unselected group of major abdominal emergency surgery [15]. In addition, Wolted et al. described a mortality rate of 16.5% in a population of 138 patients who underwent an emergency abdominal surgery [16]. Therefore, NIR is a safe technique without an increase of grade 3–5 complications according to Dindo–Clavien-Classification [14], acknowledging that almost half of our patients had an ASA score superior or equal to 3.

Moreover, this study shows that NIR angiography is a quick (median time of the procedure was less than 3 min) and easily performed procedure, even by less experienced surgeons (half of the procedures have been realized by registrars).

However, despite NIR angiography, we reported two cases of anastomosis leak in our study. NIR angiography in an emergency setting does not seem to have the same impact as in elective situation, where NIR allows to decrease anastomotic leak rate [17–20]. One explanation for the occurrence of those leaks is the questionable indication for anastomosis for those two patients with metastatic occlusive colon cancers, who were in poor general conditions under noradrenaline support and in a septic condition. Indication for anastomosis should be raised independently of the perfusion status, but considering local environment and factors as well.

Otherwise, we observed that NIR angiography was performed to check the final result of the anastomosis in only 10 of the 20 cases. After bowel resection and NIR test to control bowel vascularization, on-call surgeons seemed to have little doubt about anastomosis perfusion unlike organ viability or section margins. The feeling of the surgeon was confirmed by the NIR test: it led to a significant change of strategy only when this tool was used to assess organ viability before bowel division.

While bringing new and promising data, this study has some limitations that deserve a comment. First, it is a single-center observational study. These results should be confirmed by prospective randomized controlled trials. Then, interpretation of the NIR angiography imaging remains mainly subjective, as there is no quantification of the signal and the evaluation is performed comparing healthy bowel to the zone of interest. A standardized grad scale or quantification of the signal need to be developed to improve objectivity. However, even with the obvious bias, we found a good inter-observer reproducibility among 17 different on-call surgeons.

## Conclusion

NIR angiography is quick procedure, which allows not only the assessment of the organ viability, but also to determine the extent of the resection. NIR assessment during emergency abdominal surgery could improve patient outcomes and facilitate decision making even for less experienced surgeons, especially in front of difficult cases.

## Electronic supplementary material

Below is the link to the electronic supplementary material.

Supplementary material 1: Image 1: NIR angiography in a patient with small bowel volvulus: initially planned for bowel resection but finally it was not necessary. 1: standard view with a 30° laparoscope: doubt about organ viability because of bowel color. 2: NIR test: signal onset along the entire bowel segment. 3: NIR angiography coupled with enhanced reality for better visualization of the perfusion

Supplementary material 2: Image 2: NIR angiography in a septic patient: despite its apparent poor perfusion, this bowel segment could be spared. 1: standard view with a 30° laparoscope. 2: NIR test. 3: NIR angiography coupled with enhanced reality

Supplementary material 3: Image 3: NIR Angiography before section to assess perfusion of the pre-selected area. 1: Standard view with preliminary marking of the section area (surgical forceps). 2: NIR test: clear cut off point on the bowel. 3: NIR angiography coupled with enhanced reality: for better visualization of the cut off point
