# Supplementary figures and images for: Does near-infrared (NIR) fluorescence angiography modify operative strategy during emergency procedures?
Source: Surg Endosc. 2018 May 16;32(10):4351–6. doi: 10.1007/s00464-018-6226-9 (PMC6132899; doi:10.1007/s00464-018-6226-9)

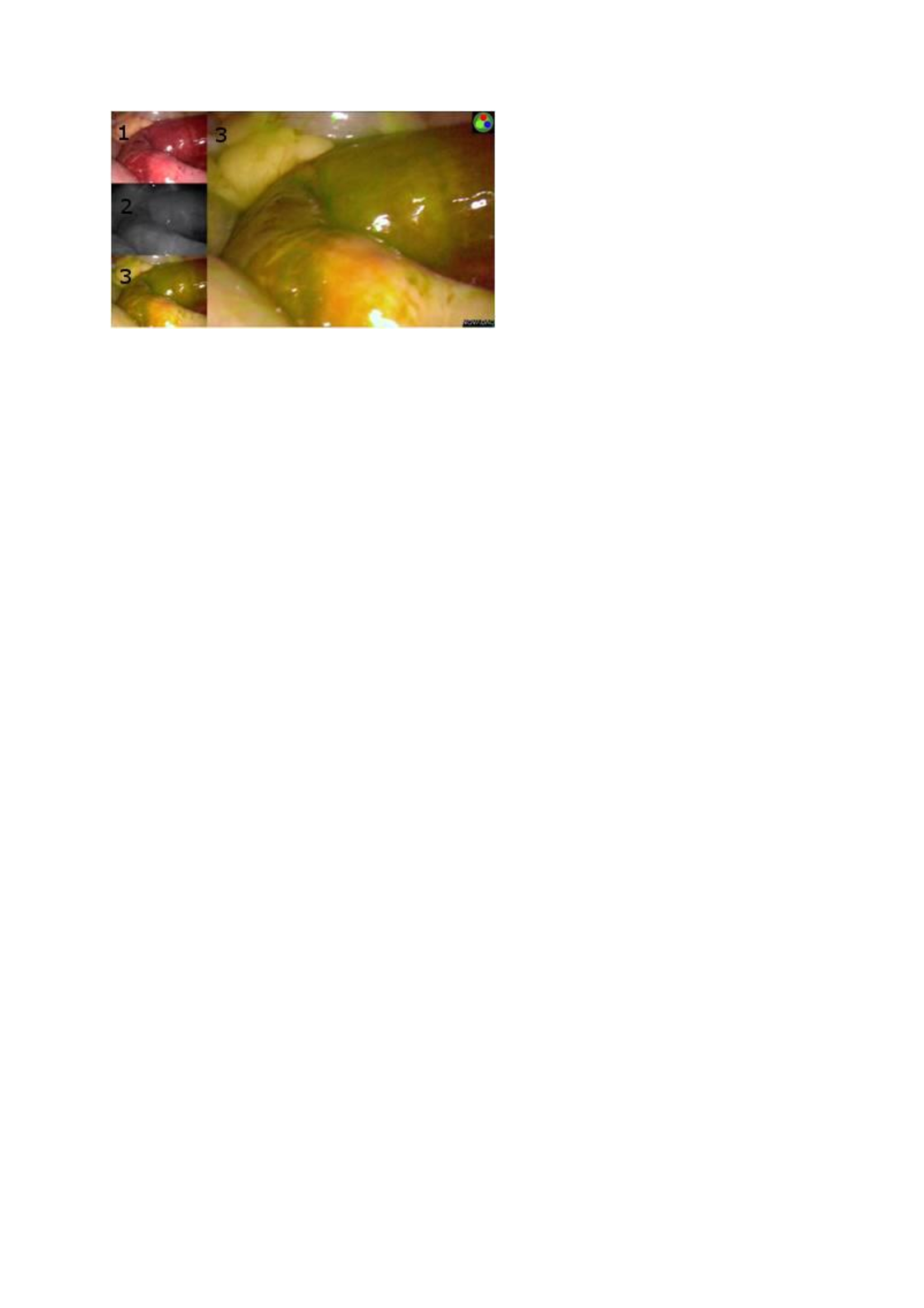

Supplement: Supplementary file 1 — Supplementary material 1: Image 1: NIR angiography in a patient with small bowel volvulus: initially planned for bowel resection but finally it was not necessary. 1: standard view with a 30° laparoscope: doubt about organ viability because of bowel color. 2: NIR test: signal onset along the entire bowel segment. 3: NIR angiography coupled with enhanced reality for better visualization of the perfusion [file 464_2018_6226_MOESM1_ESM.jpg]

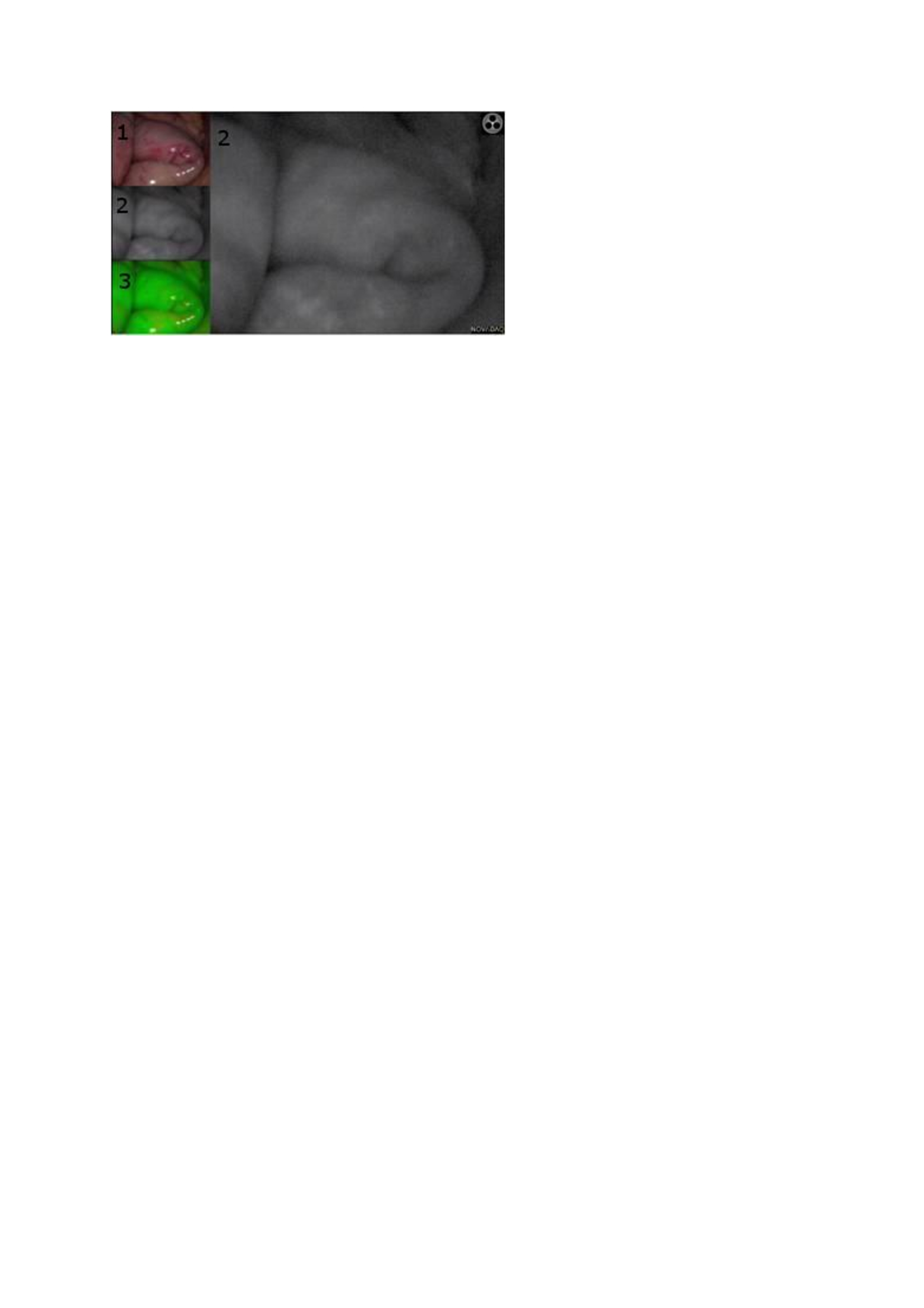

Supplement: Supplementary file 2 — Supplementary material 2: Image 2: NIR angiography in a septic patient: despite its apparent poor perfusion, this bowel segment could be spared. 1: standard view with a 30° laparoscope. 2: NIR test. 3: NIR angiography coupled with enhanced reality [file 464_2018_6226_MOESM2_ESM.jpg]

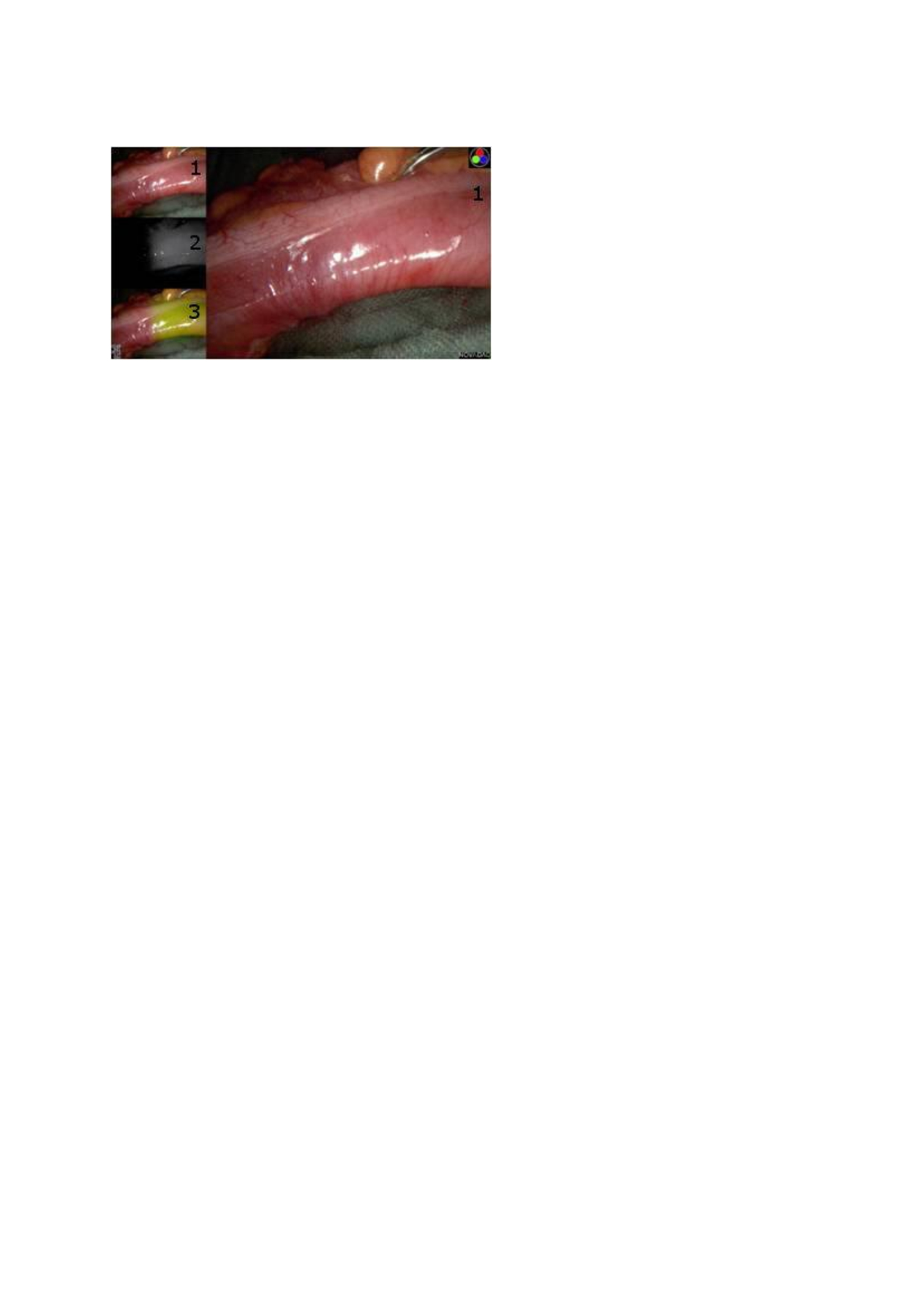

Supplement: Supplementary file 3 — Supplementary material 3: Image 3: NIR Angiography before section to assess perfusion of the pre-selected area. 1: Standard view with preliminary marking of the section area (surgical forceps). 2: NIR test: clear cut off point on the bowel. 3: NIR angiography coupled with enhanced reality: for better visualization of the cut off point [file 464_2018_6226_MOESM3_ESM.jpg]
